# HSV-mediated expression of interleukin-4 in dorsal root ganglion neurons reduces neuropathic pain

**DOI:** 10.1186/1744-8069-2-6

**Published:** 2006-02-17

**Authors:** Shuanglin Hao, Marina Mata, Joseph C Glorioso, David J Fink

**Affiliations:** 1 Department of Neurology, University of Michigan Health System, 1500 E. Medical Center Drive, Room 1914 TC, Ann Arbor, Michigan, 48109-0316, USA; 2 Department of Molecular Genetics and Biochemistry, University of Pittsburgh, 200 Lothrop St., Pittsburgh, Pennsylvania, 15261, USA; 3 VA Ann Arbor Healthcare System, 2215 Fuller Road, Ann Arbor, Michigan, 48105, USA

## Abstract

**Background:**

To examine the role of inflammatory mediators in neuropathic pain, we used a replication-defective genomic herpes simplex virus (HSV)-based vector containing the coding sequence for the anti-inflammatory peptide interleukin (IL)-4 under the transcriptional control of the HSV ICP4 immediate early promoter, vector S4IL4, to express IL-4 in dorsal root ganglion (DRG) neurons in vivo.

**Results:**

Subcutaneous inoculation of S4IL4 in the foot transduced lumbar DRG to produce IL-4. Transgene-mediated expression of IL-4 did not alter thermal latency or tactile threshold in normal animals, but inoculation of S4IL4 1 week after spinal nerve ligation (SNL) reduced mechanical allodynia and reversed thermal hyperalgesia resulting from SNL. Inoculation of S4IL4 1 week before SNL delayed the development of thermal hyperalgesia and tactile allodynia, but did not prevent the ultimate development of these manifestations of neuropathic pain. S4IL4 inoculation suppressed non-noxious-induced expression of c-Fos immunoreactivity in dorsal horn of spinal cord and reversed the upregulation of spinal IL-1β, PGE2, and phosphorylated-p38 MAP kinase, characteristic of neuropathic pain.

**Conclusion:**

HSV-mediated expression of IL-4 effectively reduces the behavioral manifestations of neuropathic pain, and reverses some of the biochemical and histologic correlates of neuropathic pain at the spinal level.

## Background

Neural-immune interactions play a key role in the pathogenesis of neuropathic pain. Partial nerve injury results in the release of proinflammatory cytokines interleukin (IL)-1β, IL-6, and tumor necrosis factor alpha from activated Schwann cells, endothelial cells, and macrophages in nerve [1] to produce direct effects on nociceptive activity [2]. Peripheral nerve damage also activates microglia and astrocytes in spinal cord to release the same proinflammatory cytokines in dorsal horn [3-6], resulting in central sensitization [7,8] and an exaggerated pain response characteristic of neuropathic pain [9-11].

IL-4 is a prototypical anti-inflammatory cytokine that modulates macrophage activity through global suppression of proinflammatory cytokines [12-14], in addition to pleiotropic effects on the development of immune cells and the immune response [15]. The broad spectrum of IL-4 action makes it an attractive candidate for suppressing cytokine activation in neuropathic pain, but the short half-life of the peptide and its pleiotropic effects on immune responsiveness preclude the use of systemic IL-4 for the treatment of pain.

Gene transfer provides the opportunity to produce the release of short-lived peptides in restricted distributions in the nervous system [16,17]. Transduction of sensory neurons of the dorsal root ganglion (DRG) by footpad inoculation of recombinant herpes simplex virus (HSV)-based vectors can be used to achieve the local release of neuropeptides from the central and peripheral terminals of primary afferents in dorsal horn of spinal cord [18-20]. Gene transfer to DRG by HSV vectors coding for proenkephalin produces an anti-hyperalgesic effect in models of acute thermal nociception, provides an anti-nociceptive effect in subacute inflammatory and neuropathic pain, and reduces both nociceptive behavior and joint destruction in a rodent model of arthritis [18-21]. HSV-mediated release of proenkephalin or the glial cell line-derived neurotrophic factor from DRG neurons provides an analgesic effect in the spinal nerve ligation (SNL) model of neuropathic pain [22,23], and an HSV vector expressing glutamic acid decarboxylase produces gamma-aminobutyric acid to provide an anti-nociceptive effect in the rodent model of chronic spinal cord injury pain [24].

To examine the role of inflammatory cytokines in the SNL model of neuropathic pain, we used an HSV vector expressing the anti-inflammatory cytokine IL-4 delivered to the DRG by subcutaneous inoculation in the foot. We found that HSV-mediated expression of IL-4 reduced mechanical allodynia and thermal hyperalgesia and reduced the induction of c-Fos-like immunoreactivity (Fos-LI) by non-noxious touch, and also blocked the increase in expression of IL-1β, PGE2, and phosphorylated p38 (p-p38) in the spinal cord after SNL.

## Results

### S4IL4 produces IL-4 in vitro and in vivo

**Figure 1:**
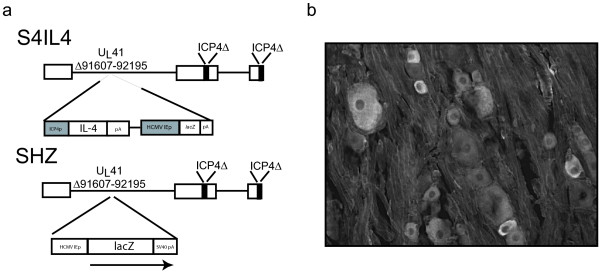
(a) Schematic representation of vector S4IL4. The control vector SHZ was identical to S4IL4, except that the inserted cassette contained the *E. coli *lacZ reporter gene under the control of the HCMV IEp in the tk locus. (b) Immunofluorescent staining shows IL-4 expression in large and small neurons in the DRG 1 week after subcutaneous inoculation of vector S4IL4.

Transduction of primary DRG neurons in culture with S4IL4 (Figure 1a) at a multiplicity of infection (MOI) of 1 resulted in the production and release of substantial amounts of IL-4 into the medium as detected by ELISA (110 pg/ml over 24 hrs from cells infected with S4IL4 compared to 10 pg/ml from cells infected with control vector SHZ). Rats inoculated subcutaneously in the foot with 30 μl of S4IL4 containing 4 × 10^8 ^pfu 1 week after L5 SNL and sacrificed 2 weeks later had 23.7 pg IL-4 per DRG, compared to no detectable IL-4 in SHZ-inoculated animals. Immunofluorescent staining of DRG 1 week after vector inoculation revealed that IL-4 immunoreactivity was found in both large and small neurons in the DRG (Figure 1b).

### Anti-nociceptive effect of inoculation of S4IL4

**Figure 2:**
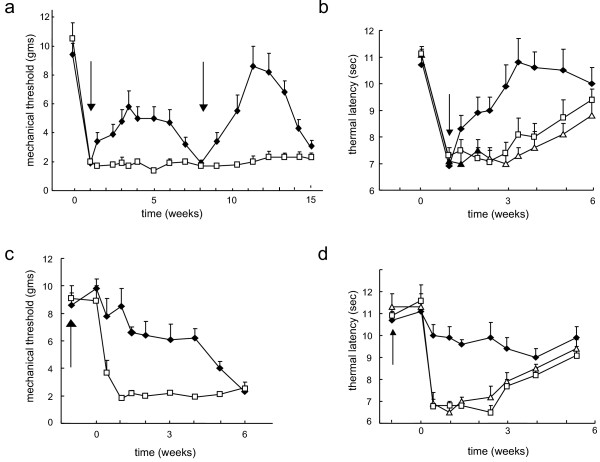
Antinociceptive effect of S4IL4 in rats with neuropathic pain resulting from SNL. Filled diamonds – S4IL4; open squares – SHZ; open triangles – vehicle control. Mechanical allodynia is identical in vehicle control and SHZ-inoculated animals [21]. Values presented as mean ± SEM, n = 6–8 animals per group. Arrows indicate time of vector inoculation. X-axis: weeks after SNL. In each case, *P *< 0.01 comparing S4IL4-inoculated to control, using the general linear model (GLM) for repeated measures. (a) Mechanical allodynia in animals inoculated with vector 1 week after SNL. (b) Thermal hyperalgesia in animals inoculated with vector 1 week after SNL. (c) Mechanical allodynia in animals inoculated with the vector 1 week prior to SNL. (d) Thermal hyperalgesia in animals inoculated with the vector 1 week prior to SNL.

Vector-mediated IL-4 production did not affect normal sensory function. Unoperated animals inoculated with either S4IL4 or SHZ showed no change in mechanical threshold or withdrawal latency to thermal stimulus compared to animals without vector inoculation over the course of 3 weeks (data not shown). L5 SNL resulted in tactile allodynia manifested by a decrease in withdrawal threshold to tactile stimuli from 11 ± 1 to 1.9 ± 0.1 g. Animals inoculated with S4IL4 1 week after SNL showed a statistically significant reduction in mechanical allodynia that was continuous and sustained. This was first observed in animals tested 3 d after inoculation and for 5 weeks, peaking at 2 weeks after the inoculation (Figure 2a). By 6 weeks after inoculation, the effect of vector inoculation was no longer apparent and the mechanical threshold of the S4IL4-injected rats was indistinguishable from that of SHZ-inoculated controls (Figure 2a). Reinoculation of S4IL4 into the foot after the anti-allodynic effect had waned resulted in reestablishment of the anti-allodynic effect, which appeared to be of greater magnitude than the original anti-allodynic effect with a similar duration. Vector-mediated IL-4 expression also reduced thermal hyperalgesia. SNL reduced the mean withdrawal latency to radiant heat from 11 ± 0.4 to 7 ± 0.3 s, a phenomenon that gradually resolved spontaneously over the course of 5 weeks (Figure 2b). Inoculation with S4IL4 but not SHZ resulted in a significant reversal of thermal hyperalgesia compared to vehicle-injected animals (Figure 2b). The effect of vector-mediated transgene expression on thermal hyperalgesia was statistically significant by 1 week and maximal by 2 weeks after inoculation.

Previous studies have suggested that proinflammatory cytokines may play a role in the initiation of neuropathic pain [3-6]; therefore, we were interested to determine whether expression of IL-4 prior to the time of spinal nerve injury would prevent the development of neuropathic pain. A set of animals was inoculated with the vector 1 week prior to SNL, preventing both mechanical allodynia and thermal hyperalgesia (Figures 2c, 2d). The magnitude of the anti-allodynic effect of prior inoculation of the vector was greater than that seen with a single inoculation performed 1 week after SNL. Nonetheless, by 5 weeks after SNL, the anti-allodynic effect of the vector was substantially reduced, and by 6 weeks after inoculation the S4IL4-inoculated animals were indistinguishable from the SHZ-inoculated controls. These results suggest that IL-4 served to suppress pain-related behaviors, but did not prevent initiation of neuropathic pain. Prior inoculation of the vector also prevented thermal hyperalgesia after the SNL, but because control animals recover by 4–5 weeks, the duration of the effect of vector pre-inoculation on thermal hyperalgesia could not be assessed.

### Effect of S4IL4 inoculation on Fos-LI in the spinal cord

**Figure 3:**
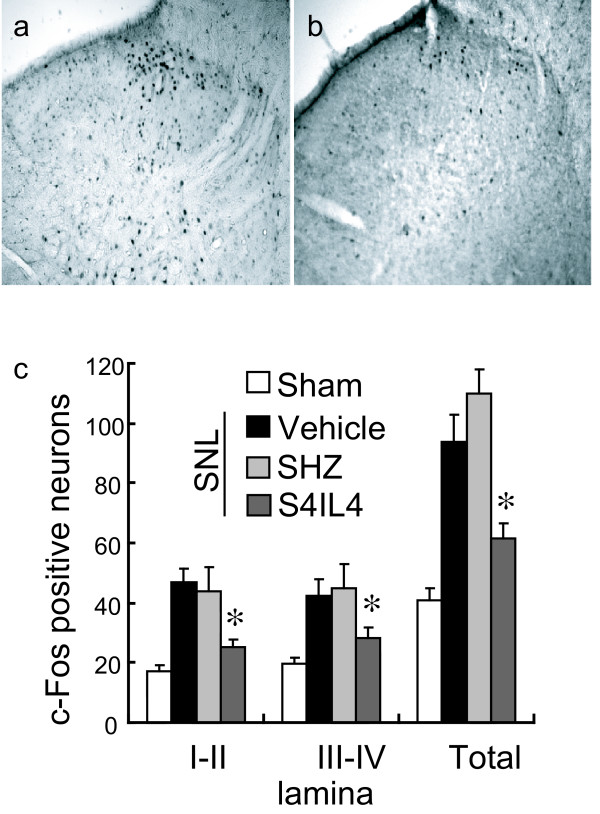
Fos-LI was induced 3 weeks after operation using 10 min of gentle tactile stimulation; animals were sacrificed 2 h later. Rats inoculated with SHZ (a) 1 week after SNL show a marked increase in Fos-LI-positive neurons compared with those inoculated with S4IL4 (b). (c) Quantitative distribution of Fos-LI neurons in laminae of the L4–5 dorsal horn. Results are expressed as the mean ± SEM numbers of Fos-LI neurons per section (*P < 0.05 compared to SHZ-inoculated; *n *= 5, ANOVA).

Non-noxious touch-induced expression of c-Fos in the dorsal horn provides a histologic marker of nociceptive processing in the dorsal horn (Figures 3a, 3b). Animals inoculated with S4IL4 showed a substantial reduction in the number of c-Fos immunoreactive neurons after non-noxious touch in both superficial (I-II) and deeper (III-IV) laminae of the dorsal horn compared to vehicle or SHZ-inoculated SNL animals (Figure 3c).

### Effect of S4IL4 inoculation on spinal IL-1β, PGE2, and p-p38

**Figure 4:**
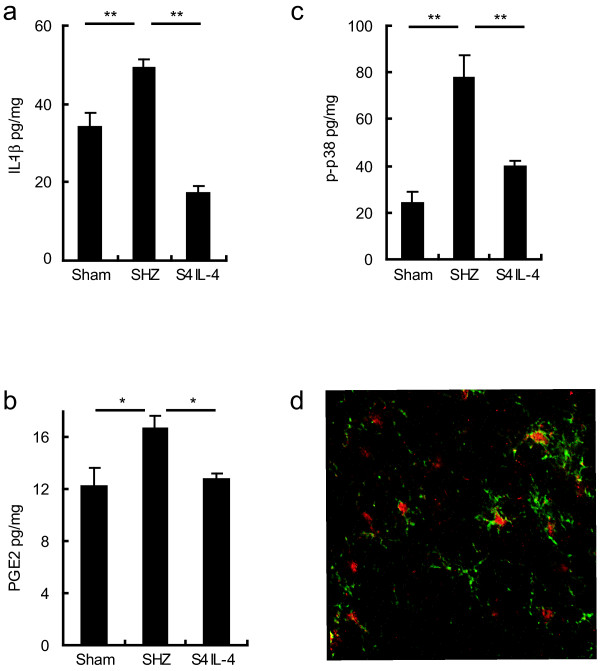
IL-1β, PGE2, and p-p38 expression in the spinal cord dorsal horn in neuropathic rats. Rats were given gentle tactile stimulation to left paw for 10 min 3 weeks after SNL or sham surgery. Ipsilateral dorsal horn was harvested 2 h after gentle stimulation. Inoculation of S4IL4 but not control vector significantly suppressed the increase in levels of IL-1β (a), PGE2 (b), and p-p38α (c) (all values in pg/mg wet weight of tissue; *P < 0.05, **P < 0.01, ANOVA, post-hoc Scheffe test). (d) Double immunofluorescence demonstrates colocalization of p-p38 (red) with the microglial marker OX-42 (green) in dorsal horn 3 weeks after SNL. P-p38 did not colocalize with NeuN in spinal dorsal horn (data not shown).

SNL significantly increases levels of IL-1β, PGE2, and p-p38 in dorsal horn of spinal cord ipsilateral to the nerve injury. Animals inoculated with S4IL4 1 week after SNL showed a substantial reduction in the levels of IL-1β (Figure 4a) and PGE2 (Figure 4b) compared to animals inoculated after SNL with control vector SHZ. The level of p-p38α increased dramatically after SNL; this increase was prevented in animals inoculated with S4IL4 (Figure 4c). To identify the cell types that expressed p-p38 after SNL, we performed double-label immunostaining of p-p38 with NeuN (neurons) and OX-42 (CD-11b, microglia). Most of the p-p38 immunostaining appeared to be in the nuclei of microglia, based on the colocalization of p-p38 immmunoreactivity with the marker OX-42 (Figure 4d). p-ERK was also increased in the spinal horn of rats following SNL, but this increase was not suppressed in animals inoculated with S4IL4 (data not shown).

## Discussion

There are 3 principal results from the current study. First, the HSV-based vector S4IL4 produced IL-4 in vitro, and in vivo in DRG neurons following transfection by subcutaneous inoculation. Second, IL-4 released from DRG neurons transduced with S4IL4 in vivo reduced pain-related behavior, which correlated with a reduction in non-noxious touch-induced c-Fos expression in dorsal horn in the SNL model of neuropathic pain. Finally, S4IL4-mediated IL-4 release decreased the levels of IL-1β and PGE2 in dorsal horn, and reduced the phosphorylation of spinal p-p38.

The role of inflammatory cytokines in the development of inflammatory (nociceptive) pain has been recognized for some time [25]. More recent evidence has demonstrated that proinflammatory cytokines, including IL-1β, induce a long-term alteration of synaptic transmission in the central nervous system, and that spinal expression and release of these cytokines plays a critical role in the development and maintenance of neuropathic pain of peripheral origin [3-6]. Inflammatory cytokines increase in nerve after peripheral nerve injury as well [26], and several different antagonists of proinflammatory cytokine effects can reverse allodynia in the neuropathic pain model in rats [5,6,27].

IL-4 is a prototypical anti-inflammatory cytokine that is known to increase IL-1 receptor antagonist mRNA and protein, and to suppress the expression of IL-1β mRNA and IL-1β protein in vitro [28]. IL-4 directly inhibits the induction of nitric oxide synthase and levels of cyclooxygenase 2, the enzymes responsible for the production of nitric oxide and PGE2, respectively [29]. In models of inflammatory pain, intraplantar administration of IL-4 peptide reduces pain caused by subsequent intraplantar injection of carageeninin, bradykinin or TNFα [30], and itraperitoneal administration of IL-4 peptide produces a transient reduction in pain-related behavior, measured by the writhing response in mice and by zymosan-induced knee joint incapacitation in rats [31]. In the experiments that are the subject of the current report, we have demonstrated that transgene-mediated expression of IL-4 in DRG effectively reduces nocifensive behaviors and blocks expression of IL-1β in spinal cord, in the neuropathic pain model induced by the peripheral nerve injury (SNL).

Activation of mitogen-activated protein kinases, including extracellular signal-related kinase and p38, contribute to the development and maintenance of pain hypersensitivity through transcription-dependent and transcription-independent pathways [32-34]. Activated p38 participates in the maintenance of inflammatory heat hyperalgesia [35], p-p38 is increased in non-neuronal cells in the spinal cord after nerve injury, and in vitro application of IL-1β induces phosphorylation and activation of p38 mitogen-activated protein kinase. IL-4 produced by subcutaneous inoculation of S4IL4 suppressed expression of p-p38 but not pERK in the dorsal horn.

The time course of the analgesic effect of vector-mediated IL-4 observed in this study is similar to the time course on the analgesic effects that we have observed in experiments using HSV-mediated gene transfer to DRG to produce the release of enkephalin [20,21]and GABA [24,36]. We believe it is most likely that the transient time course of the analgesic effect reflects the time course of transgene expression characteristic of the viral immediate early gene promoters [37] that we have employed in these vectors; the HSV ICP4 promoter in vector S4IL4 in the current study, and the human cytomegalovirus immediate early promoter (HCMV IEp) in the enkephalin- and GABA-producing vectors. In the current study as in those previous studies we have observed that reinoculation of the vector after the effect has waned produces an effect that is at least equivalent to, and appears to be greater than, the effect produced by the initial inoculation of the vector. The design of the studies does not allow for a direct statistical comparison of the effect of the second inoculation to that produced by the first; the observation that the effectiveness of the vector is not reduced following reinoculation suggests that there is no significant immune response to the initial vector inoculation in these models.

The observation that intrathecal administration of an anti-IL-6 antibody and IgG [38], the anti-inflammatory cytokine IL-10 [39], or a combination of IL-1 receptor antagonist and the soluble tumor necrosis factor receptor [40] in models of peripheral neuropathic pain reduce nocifensive behaviors, supports the contention that spinal expression of inflammatory mediators plays a role in the spinal sensitization characteristic of chronic neuropathic pain. A peripheral action of proinflammatory cytokines in the pathogenesis neuropathic pain have also been defined in model systems [41]. In the gene transfer experiments reported herein we have not established the locus of action of the HSV-mediated IL-4. IL-4 produced in the DRG is transported both centrally (towards the spinal cord) and peripherally (towards the skin) in the bipolar axon of the sensory neuron, and the experiments performed do not allow us to distinguish the effects of IL-4 in the nerve from effects that occur at the level of the spinal cord. Gene transfer to meninges by intrathecal inoculation of IL-2 using an adenovirus vector [42] or IL-10 using an adeno-associated gene transfer vector [43] reduces pain in the chronic constriction injury model of neuropathic pain. Compared to adeno-associated viral vector gene transfer to the meninges, HSV-mediated gene transfer has 2 advantages: restricted release of the peptide into the spinal cord via distribution into central projections of the transduced neurons provides an anti-inflammatory effect restricted to the region where spinal inflammation related to pain occurs, and axonal transport of the peptide from the DRG into nerve may provide additional reduction in pain-generating mechanisms. Wherever the effect occurs, effective treatment of chronic neuropathic pain will need to be directed at the prevention or reversal of the proinflammatory state in the dorsal horn. In this regard, HSV-mediated release of anti-inflammatory cytokines may be particularly useful in the development of novel treatments for human regional neuropathic pain syndromes.

## Materials and methods

### Construction of the IL-4-expressing HSV vector

S4IL4 (Figure 1a) was generated as described [44] by Cre-lox recombination of a plasmid containing the HSV ICP4 promoter, murine IL-4, an HCMV-β-galactosidase expression cassette and a lox recombination site [45] into the thymidine kinase locus of d120 [46] which is a KOS strain HSV with a deletion in both copies of the immediate-early ICP4 [47]. Recombinants were screened by X-gal staining for β-galactosidase expression and purified by 3 rounds of limiting dilution. Viral constructs were verified by Southern blot analysis, propagated, and purified as described [48]. Control vector SHZ (Figure 1a) is similar to S4IL4, but contains only lacZ under the control of the human cytomegalovirus immediate-early promoter in the thymidine kinase locus.

### Expression of IL-4 in vitro

DRG neurons from 17-day-old embryonic rats were cultured on polylysine-coated coverslips in B27/Neurobasal medium, Glutamax II, AlbuMAX, and nerve growth factor (NGF) supplementation. At 14 d in vitro, the cells were infected for 1 h with either S4IL4 or SHZ at an MOI of 1, and the medium changed to fresh medium without NGF. Twenty-four hrs postinfection the medium was collected and the amount of IL-4 determined by ELISA (R&D Systems, Minneapolis, MN).

### Experimental animals and surgical procedures

Male Sprague-Dawley rats weighing 225–250 g were used. All housing conditions and experimental procedures were approved by the Institutional Animal Care and Use Committee. Under chloral hydrate anesthesia, one-third of the left L6 transverse process was removed and nerve injury was produced by tight ligation of the L5 spinal nerve using 6-0 silk suture [49]. Sham control animals were treated identically, except that the spinal nerve was not ligated. Rats with SNL exhibited a change in the ipsilateral foot position and displayed a "guarding behavior" of ipsilateral hind limb, but animals displaying motor dysfunction of the proximal hind limb after operation were excluded from further analysis. All the animals exhibited normal weight gain, toenail growth, and level of activity. One week after the operation the rats were inoculated subcutaneously in the plantar surface of the left hind paw with 30 μl of S4IL4 or control vector SHZ at a concentration of 4 × 10^8 ^plaque-forming units/ml. There was no evidence of distress or abnormally aggressive behavior in any of the animals after inoculation with HSV vector.

### Behavioral testing

#### Mechanical allodynia

Rats were placed in transparent plastic cubicles on a mesh floor for an acclimatization period of at least 30 min. Mechanical allodynia induced by L5 SNL was determined by assessing paw withdrawal to von Frey hairs of graded tensile strength. A series of calibrated von Frey filaments (0.4, 0.7, 1.2, 1.5, 2.0, 3.6, 5.5, 8.5, 11.8, and 15.1 g) were presented serially to the hind paw in ascending order of strength, with each filament applied for 6 s with sufficient force to cause slight bending against the paw. A positive response, defined as rapid withdrawal and/or licking of the paw immediately upon application of the stimulus, was followed by application of the next finer von Frey filament. After a negative response, the next higher von Frey filament was applied. Animals that did not respond to a pressure of 15.1 g were assigned to this cut off value. The tactile stimulus producing a 50% likelihood of withdrawal was determined using the up-down method [50,51].

#### Thermal hyperalgesia

The latency to hind paw withdrawal from a thermal stimulus was determined by exposing the plantar surface of the hind paw to radiant heat using a modified Hargreaves thermal testing device [52]. Briefly, rats were placed in individual enclosures on a glass plate maintained at 30°C and a radiant thermal stimulus was positioned underneath the glass plate directly under the hind paw. Activation of the bulb simultaneously activated a timer, and both were immediately turned off by paw withdrawal or at the 20 s cut-off time.

### Immunohistochemistry

Expression of c-Fos was induced by gentle touch applied once every 4 s for 10 min, as was previously described [53,54]. Each stimulus was moved from the middle of the foot to the distal footpad over the course of 2 s in a manner that would not elicit a flexion reflex in normal rats. Two hours following completion of stimulation, the animals were deeply anesthetized with chloral hydrate and perfused through the heart with 300 ml saline followed by 500 ml of 4% paraformaldehyde in 0.1 M phosphate buffered saline (PBS). The spinal cord was post-fixed for 4 h, cryo-protected in 30% sucrose in PBS, and 35 μm transverse sections of L4–L5 segments incubated overnight at 4°C with the primary antibody (anti-c-Fos, 1:500 or anti-pERK1/2, 1:200; Santa Cruz Biotechnology, Inc., Santa Cruz, CA, or anti OX-42, 1:1000, BD Biosciences, San Jose, CA). This was followed by the secondary antibody (biotinylated goat anti-rabbit IgG, 1:100; Vector Laboratories, Burlingame, CA) and detected using the Vectastain Elite ABC Kit (Vector Laboratories) as described [23,55]. The sections were developed with diaminobenzidine (DAB Substrate Kit; Vector Laboratories) for 2 to 6 min, then washed with PBS before being mounted onto gelatin-coated slides, dehydrated, and coverslipped. Ten sections from each rat were randomly selected and the expression of Fos-LI and pERK1/2 evaluated by a blinded observer counting the number of immunopositive neurons in the gray matter of ipsilateral spinal cord.

### Immunofluorescent staining

Spinal sections prepared as described above were incubated with the primary antibody (anti-p-p38, 1:150, Cell Signaling Technology, Beverly, MA) overnight at 4°C, followed by a CY-3 conjugated secondary antibody for 1 h at room temperature. For double-label fluorescence, spinal sections were incubated with a mixture of polyclonal anti-p-p38 and anti-NeuN (1: 500, Chemicon International, Temecula, CA) or OX-42 (BD Pharmigen 1:500, BD Biosciences, San Jose, CA) over 2 nights at 4°C, followed by a mixture of CY3 or Alexafluor 488 conjugated secondary antibodies (Molecular Probes, Eugene, OR) for 1 h at room temperature. The stained sections were examined using an Olympus Fluoview BX61 confocal microscope (Olympus America, Melville, NY).

### ELISA

Quantitative evaluation of IL-4 protein expression in vivo was determined by ELISA. One week after selective L5 SNL animals were inoculated with either S4IL4 or SHZ as described above. Two weeks after inoculation, the L4 DRG were removed, frozen on dry ice, and stored at -80°C. The tissue was minced and placed in 0.3 ml of ice-cold homogenization buffer (50 mM NaCl, 10 mM Tris, 2.5 mM MgCl_2_, pH 7.4) containing 1 protease inhibitor cocktail tablet (Roche, Mannheim, Germany) per 50 ml, as described previously [23]. To determine the amount of IL-1β, PGE2, and p-p38α in other animals the ipsilateral dorsal quadrant of lumbar spinal cord was processed in an identical fashion and peptide levels determined using specific ELISA kits (R&D Systems).

### Data analysis

All data presented as mean ± SEM. The statistical significance of differences between groups was evaluated by ANOVA, using Scheffe's post-hoc test. For repeated measures of behavioral function, parametric statistics, using the general linear model (GLM) for repeated measures were used to identify significant effects of treatment condition on the behavioral measure of neuropathic pain. The results were examined for a main effect of treatment group. All statistical analyses were performed using the software package, SPSS 13.0 for Windows (SPSS Inc., Chicago, Illinois).

## Conclusion

These experiments demonstrate that expression of IL-4 in DRG neurons, achieved by HSV-mediated gene transfer in vivo, reduces mechanical allodynia and thermal hyperalgesia in the SNL model of neuropathic pain. Inoculation of S4IL4 1 week before SNL delayed the development of thermal hyperalgesia and tactile allodynia, but did not prevent manifestations of neuropathic pain. S4IL4 inoculation suppressed non-noxious-induced expression of c-Fos immunoreactivity in dorsal horn of spinal cord, and reversed the upregulation of spinal IL-1β, PGE2, and p-p38 MAP kinase, characteristic of neuropathic pain.

## List of abbreviations used

DRG dorsal root ganglion

Fos-LI c-Fos-like immunoreactivity

HSV herpes simplex virus

IL interleukin

MOI multiplicity of infection

NGF nerve growth factor

PBS phosphate buffered saline

p-p38 phosphorylated p-38

SNL spinal nerve ligation

## Competing interests

JCG is owns stock and is employed as a consultant by Diamyd. SH, MM, and DJF declare that they have no competing interests.

## Authors' contributions

JCG provided the IL4-expressing vector. SH carried out the studies described. MM supervised the experiments. DJF and MM designed the experiments and wrote the manuscript. All authors read and approved the final manuscript.
